# Use of the Brief-BESTest partially instrumented with accelerometry to detect balance deterioration in middle-age

**DOI:** 10.1007/s40520-024-02868-7

**Published:** 2024-11-09

**Authors:** Guy Baranes, Roee Hayek, Itai Gutman, Silvi Frenkel-Toledo, Shmuel Springer

**Affiliations:** 1 Faculty of Health Sciences, Department of Physical Therapy, The Neuromuscular & Human Performance Laboratory, Ariel University, Ariel, Israel; 2 Faculty of Health Sciences, Department of Physical Therapy, Brain and Motor Behavior Laboratory, Ariel University, Ariel, Israel; 3 Research Associate Canadian Center for Activity and Ageing, University of Western Ontario, London, ON N6A 3K7 Canada

**Keywords:** Aging, Middle-age, Balance assessment, Sway, Brief-BESTest, Accelerometry balance assessment

## Abstract

**Background:**

Most standardized balance tests cannot detect subtle balance deterioration in middle age, or identify those at higher risk for accelerated balance decline due to a ceiling effect.

**Aims:**

To determine whether the Brief Balance Evaluation Systems Test (Brief-BESTest), partially instrumented with accelerometry, can detect balance deterioration in middle age and identify individuals with poor balance.

**Methods:**

We studied young (25.3 ± 2.3 years), early middle-aged (47.7 ± 2.6 years), and late middle-aged adults (60.6 ± 3.6 years), with 25 participants in each age group. Subjects wore an accelerometer on their lower back while performing the Brief-BESTest. Balance measurements included the Brief-BESTest total and sub-measures scores, and postural sway during the Brief-BESTest standing tasks, calculated by the 95% confidence ellipse trajectory of the center of mass (COM-95% ellipse).

**Results:**

Compared to the two middle-aged groups, young adults had better total Brief-BESTest and sub-measures scores, apart from the Stability-in-Gait sub-measure, and less postural sway during the Sensory-Orientation sub-measure. The total Brief-BESTest scores as well as the Biomechanical-Constraints and Sensory-Orientation sub-measures differed also between early and late middle-aged adults. Both the Brief-BESTest total scores and the Sensory-Orientation postural sway values demonstrated increased variation with age, allowing to identify subjects with poor balance. A moderate negative correlation (*r* = -0.43) was found between the Brief-BESTest total score and the COM-95% ellipse size, and a moderate agreement (*k* = 0.56) in identifying subjects with poor performance in the early but not the late middle age group.

**Conclusions:**

The Brief-BESTest test combined with accelerometry could be a suitable screening tool to identify middle-aged people with early balance deterioration and potentially identify those with poor balance and a possible higher risk for falls. Clinicians and policymakers can use our findings to implement balance assessment programs in patients < 65 years, leading to preventive strategies before the risk increases.

**Supplementary Information:**

The online version contains supplementary material available at 10.1007/s40520-024-02868-7.

## Introduction

Age-related deterioration of balance in older adults, associated with an increased risk of falls and negative health outcomes, has been recognized as a global problem [1, 2]. Consequently, a large body of research has focused on screening tools to assess fall risk in older individuals and the development of targeted interventions. Despite ongoing efforts, the prevalence of falls among older adults has remained relatively stable, primarily due to the increasing aging population, chronic health conditions, and persistent environmental and socioeconomic challenges. Furthermore, insufficient awareness of the importance of physical activity and underreporting of cognitive and physical impairments also contribute to this problem [3–7].

Emerging evidence indicates the importance of initiating fall risk assessment before the age of 65, allowing for implementing preventive strategies before the risk accelerates [8–10]. A recent scoping review proposed adopting the term ‘preclinical mobility limitation’ (PCML) to characterize subtle, scarcely noticeable performance changes [11]. The middle age (i.e., 45–64 years) may be an ideal period to identify individuals with early balance deterioration [12]. Up to 10–20% of people in this age group report having some difficulties with mobility, despite having only mild and common health problems such as back pain [13–15]. This statistic underscores the significance of middle age as a critical life stage, as evidenced by the estimated annual prevalence of falls, which triples from 9% in adults aged 40 to 44 years to 28% in those aged 60 to 64 years [16].

Though the deterioration of balance in middle age has been attributed to a decline in key balance regulatory systems, namely the proprioceptive [17, 18], vestibular [19, 20], visual [21, 22], and musculoskeletal systems [23–25], , it can be difficult to detect early subtle changes in balance in this population. Common clinical balance tests such as Timed Up and Go, Berg Balance Scale, Dynamic Gait Index, Functional Reach, and single limb stance have limited ability to detect such changes, as they may have a ceiling effect even in community-dwelling older people [26–28].

The Balance Evaluation Systems Test (BESTest) is a balance test consisting of 6 different domains of static and dynamic postural control [29]. To reduce its relatively long administration time (i.e., 20 to 60 min) [29, 30] and increase its applicability, alternative, shorter versions of the BESTest have been proposed [30, 31]. The Brief-BESTest is a shortened version of the BESTest consisting of six sub-measures, one item from each section of the original test [30]. The Brief-BESTest has shown good psychometric properties and high sensitivity in identifying individuals with a history of falls [30, 32, 33].

O’Hoski et al. [34], studied healthy adults over 50, including a large proportion of middle-aged participants (50–65 years), using the Brief-BESTest. They found decreased balance and increased balance score variation with age, suggesting the potential suitability of this test for assessing postural control in midlife. However, further research is needed to address gaps and confirm the suitability of the Brief-BESTest for a comprehensive assessment of balance in midlife. Comparing middle-aged individuals with young adults, and comparing age groups within the entire middle-aged range (45–65 years) will enhance understanding of aging’s effects on balance, which may begin deteriorating after 40 [35]. In addition, factors such as muscle strength and level of physical activity can affect balance and should be monitored.

Moreover, previous research has shown that technology-based assessments may eliminate the ceiling effects associated with some balance measures [36], and therefore can better detect early balance deterioration in middle age [37]. Instrumented assessment based on accelerometry data provides a simple and cost-effective method for implementing technology into balance assessment in research and clinical settings [37, 38]. Studies have demonstrated the validity of accelerometry-derived outcomes by correlating them with established gold standard balance assessment technologies, such as motion capture cameras and force plates, suggesting a practical and low-cost alternative to measuring balance [37–39]. Accelerometry-derived measures of postural sway have been shown to provide a thorough and reliable assessment of balance control, particularly concerning biomechanical and physiological characteristics [40, 41]. 

The study aimed to evaluate the utility of the Brief-BESTest and a partially instrumental assessment of sub-measures of the Brief-BESTest based on accelerometry data in detecting balance deterioration in middle-age and identifying middle-aged individuals with poor balance. We hypothesized that balance scores would differ significantly between middle-aged and young adults and that greater variance in balance performance with increasing age would allow identification of middle-aged individuals with poor balance.

## Methods

### Participants

This was a cross-sectional study with 75 participants from three age cohorts (25 subjects each) healthy young adults (YA) aged 20–30 years, healthy early middle-aged (EMA) individuals aged 45–54 years, and healthy late middle-aged (LMA) individuals aged 55–65 years. Our primary analysis focused on comparing balance scores between middle-aged and young adults. Previous studies [21, 42] have indicated that a sample size of at least 20 subjects per group would be sufficient to detect potential differences in performance between middle-aged and young adults. Additionally, a power analysis using G*power 3.1.9 [43] for a one-way ANOVA, with a power of 0.8, alpha = 0.05, and a large effect size (f = 0.4), estimated that a total sample size of *n* = 64 across the three groups (i.e., YA, EMA, and LMA) would be necessary to detect a significant main effect. To ensure a balanced number of participants in each age group, we studied a total sample of 75 participants. The target population was university students and employees as well as residents of the local town. Candidates were screened by telephone to determine eligibility for the study. Individuals were included in the study if they were between the ages of 20 to 30 or 45 to 65, lived independently in the community, and were able to walk outdoors and perform moderate to vigorous physical activities of daily living without assistance. Individuals with neurological, orthopedic, vestibular, or significant visual impairments (e.g., age-related macular degeneration, glaucoma, cataract, diabetic retinopathy) or other comorbidities that could affect balance were excluded. Written informed consent was obtained from the participants and a copy of the consent form was given to each participant.

### Procedure

Each subject participated in a single session that lasted approximately 60 min at XXX University Neuromuscular and Human Performance Laboratory, between August 2023 and January 2024. Participants were instructed to dress comfortably and wear flat shoes.

Two physical therapists collected the data for this study. They were further trained in administering and scoring the Brief-BESTest by a physical therapist who had extensive experience in administering the test to ensure consistency of scores. The existing literature on the BESTest clearly demonstrates that pre-training ensures consistency of scoring [30, 34, 44]. Moreover, to further eliminate bias related to inter-rater reliability, all physical therapists scored the first five participants and the scores for each item were then compared to verify consistency. Before assessing balance, anthropometric measures were collected, the level of physical activity was measured by the International Physical Activity Questionnaire- Short Form (IPAQ-SF) [45, 46], and general strength was quantified by the grip strength (Jamar^®^, 5030J1, Patterson Medical, Warrenville, IL, USA) of the dominant hand (i.e., the hand used for most tasks, such as writing and throwing a ball).

### Balance assessment

Participant’s balance was assessed using the Brief-BESTest [30] .The test consists six sub-measures as follows: Biomechanical-Constraints – tested by hip/trunk lateral strength; Stability-Limits – tested by functional reach forward; Anticipatory-Postural-Adjustments – tested by the ability to maintain single leg stance in each leg; Reactive-Postural-Responses – tested by compensatory lateral stepping response in each side; Sensory-Orientation – tested by stance on foam with eyes closed; and Stability-in-Gait – tested by timed up and go. Two sub-measures (Anticipatory-Postural-Adjustments and Reactive-Postural-Responses) are scored bilaterally in each leg, resulting in 8 test items. Each item scored between 0 and 3 and the maximal total score is 24 (i.e., a higher score indicates better balance performance). The tester read the instructions aloud and demonstrated each Brief-BESTest item to the participant. The participant then completed each task under the tester’s supervision to ensure safety [30].

Accelerometry data for the instrumented Brief-BESTest was recorded during the performance of the two stationary standing tasks, the Anticipatory-Postural-Adjustments (i.e., right and left single leg stance) and Sensory Orientation (i.e., a stance with eyes closed on a foam surface) sub-measures. Before testing, an accelerometer, Delsys Trigno, Delsys Inc. (Boston, MA, USA), was attached to the subject’s lower back to collect accelerometry data at a sampling rate of 148 Hz. The accelerometer recorded postural sway by the 95% confidence ellipse of each participant’s center of mass trajectory (COM-95% ellipse). The COM-95% ellipse measurement with an inertial sensor provides a valid representation of the dispersion of the COM trajectory data points [40].

Accelerometry data from the Delsys sensor were analyzed using a custom-written MATLAB program (MathWorks, Natick, MA) that measures the area of the ellipse by multiplying the linear acceleration of the anterior-posterior (AP) and medio-lateral (ML) planes. The area of the COM-95% ellipse $\:({\text{m}}^{4}\cdot\:{\text{s}}^{-2})$ was measured for each trail and plotted on an X-Y graph mapping the ML and AP planes.

### Data analysis

The normal distribution was assessed using the Shapiro–Wilk test and histograms. Quantitative variables were summarized for descriptive statistics using mean and standard deviation or median and interquartile range, depending on the distribution. Categorical variables were presented as frequencies and percentages.

Participant characteristics, including age, body mass index (BMI), maximal grip strength normalized to body weight, and IPAQ-SF scores were compared between the three age groups using one-way analysis of variance (ANOVA) or Kruskal-Wallis tests, as required. Significant between groups differences were further explored with post-hoc analysis (with Bonferroni correction) appropriately. Between groups differences in balance performance were assessed similarly. Balance performance variables included all Brief-BESTest scores (total and sub-measures) and the COM- 95% ellipse recorded during the Brief-BESTest’s Anticipatory-Postural-Adjustments and Sensory-Orientation sub-measures.

For variance testing, box plots were used to visually inspect the distribution across groups and to identify middle-aged subjects in the poor performance quartile (i.e., the first or last quartile depending on the direction of measurement). Kappa agreement was used to assess the agreement between measurements in identifying middle-aged subjects with poor performance (i.e., in the poor performance quartile). Kappa values were interpreted as follows: < 0 no agreement, 0.01–0.20 none to slight agreement, 0.21–0.40 fair agreement, 0.41–0.60 moderate agreement, 0.61–0.80 substantial agreement, and 0.81-1 almost perfect agreement [47].

Last, if any performance variables differed between young and middle-aged adults, they were tested for any significant association using Spearman’s correlation. The strength of the correlation coefficients was interpreted as follows: 0-0.10 negligible correlation, 0.1–0.39 weak correlation, 0.40–0.69 moderate correlation, 0.7–0.89 strong correlation, and 0.9-1 very strong correlation [48].

In addition, to further validate our results, we also conducted a sensitivity analysis in which background characteristics with significant differences between groups were analyzed using regression models to address their potential influence on balance performance. All statistical analyses were performed using IBM SPSS Statistics for Windows, version 27.0. Armonk, NY: IBM Corp. The significance value was set at *p* < 0.05.

## Results

Background characteristics were normally distributed, subsequently, between groups comparisons of the background characteristics were conducted using one-way ANOVA. The background characteristics of the participants are presented and compared between age groups in Table 1. BMI, maximal grip strength normalized to body weight, and total physical activity differed significantly between groups and were considered possible covariates (see Table 1). Post hoc analysis revealed that all the differences in the background characteristics were between YA to LMA or between YA and EMA with no significant differences between the middle-aged groups.

**Table 1:** Participants’ Background Characteristics

Background Characteristics	Young (*n* = 25)	Early Middle age (*n* = 25)	Late Middle age (*n* = 25)
**Age (years)**	25.28 (2.33)	47.72 (2.6)	60.56 (3.63)
Gender: Female (%)	13 (52)	15 (60)	14 (56)
**BMI (kg/m** ^**2**^ **)**	22.63 (3.19) ^**a, b**^	25.89 (4.13)	27.49 (3.39)
**Maximal grip normalized to body weight (Kg)** ^**b**^	0.57 (0.18)	0.49 (0.13)	0.44 (0.1)
Total physical activity (MET/week) *	2983 (2659) ^**a**^	1431 (1119)	1733 (1316)
IPAQ-SF time spent sitting (min/week)	3511 (1270)	3535 (1479)	3288 (1313)

Mean (standard deviation)

- All comparisons were conducted using one-way analysis of variance

- All *p* values are with Bonferroni adjustment for multiple comparisons

^**a**^ Significant differences between the young and early middle-aged groups (*p* < 0.05)

^**b**^ Significant differences between the young and late middle-aged groups (*p* < 0.05)

*The Kruskal-Wallis test was used as this parameter was not normally distributed

BMI- Body mass index, IPAQ- international physical activity questionnaire- short form, METS- metabolic equivalent for tasks

**Fig. 1:**
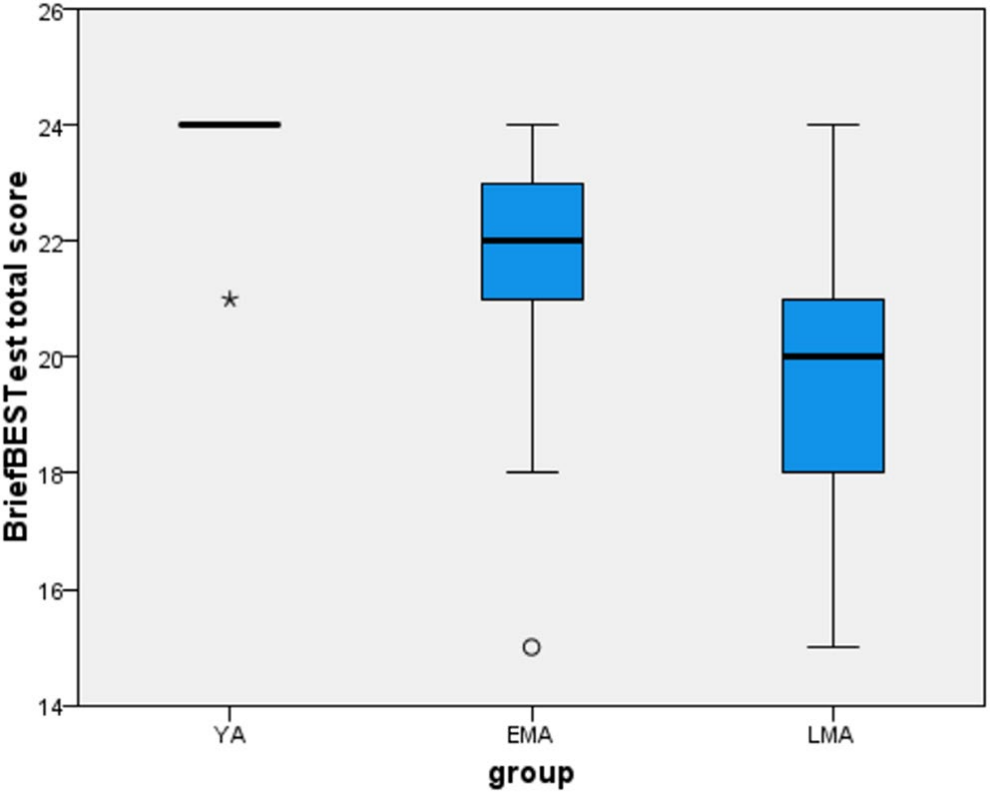
The Brief-BESTest total score in each group. Minimum and maximum values, upper and lower quartiles, and median (line inside the box) are shown. The length of the box represents the interquartile range (IQR). °Values between 1.5 to 3 IQR away from one of the two ends of the box are considered outlier values. *Values greater than 3 IQR away from one of the two ends of the box are considered extreme values. Young adults (YA), Early Middle Age (EMA), Late Middle Age (LMA)

Most balance outcomes were not distributed normally, subsequently, between groups comparisons of the balance outcomes were conducted using Kruskal-Wallis test. The scores of the Brief-BESTest total/sub-measures and the comparison between age groups are presented and plotted in Table 2; Fig. 1, respectively. The results revealed a significant difference between groups in the Brief-BESTest total score (*X*^*2*^ [2] = 45.78, *p* < 0.001). Post hoc analysis revealed significant difference between all groups with a decrease in the balance score with increasing age (YA > EMA > LMA, *p* < 0.05). Additionally, significant differences were observed in five Brief-BESTest sub-measure scores: Biomechanical-Constraints (*X*^*2*^ [2] = 33.31, *p* < 0.001), Stability-Limits (*X*^*2*^ [2] = 20.91, *p* < 0.001), Anticipatory-Postural-Adjustments (*X*^*2*^ [2] = 21.36, *p* < 0.001), Reactive-Postural-Responses (*X*^*2*^ [2] = 10.43, *p* = 0.005), and Sensory-Orientation (*X*^*2*^ [2] = 31.77, *p* < 0.001). Post hoc analysis of the five Brief-BESTest sub-measure scores are presented in Table 2.

**Fig. 2:**
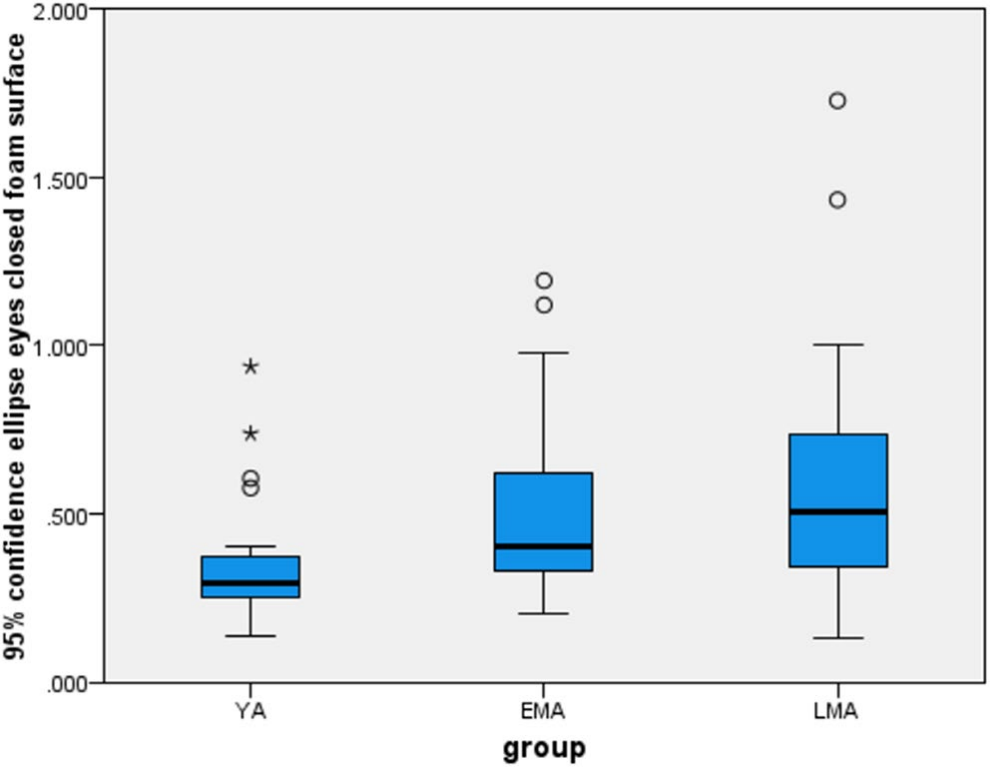
The COM-95% ellipse during the Sensory-Orientation test in each group. Minimum and maximum values, upper and lower quartiles, median and (line inside the box) are shown. The length of the box represents the interquartile range (IQR). °Values between 1.5 to 3 IQR away from one of the two ends of the box are considered outlier values. *Values that are more than 3 IQR away from one of the two ends of the box are considered extreme values. COM- center of mass, 95% ellipse- 95% Confidence ellipse, Young adults (YA), Early Middle Age (EMA), Late Middle Age (LMA)

**Fig. 3:**
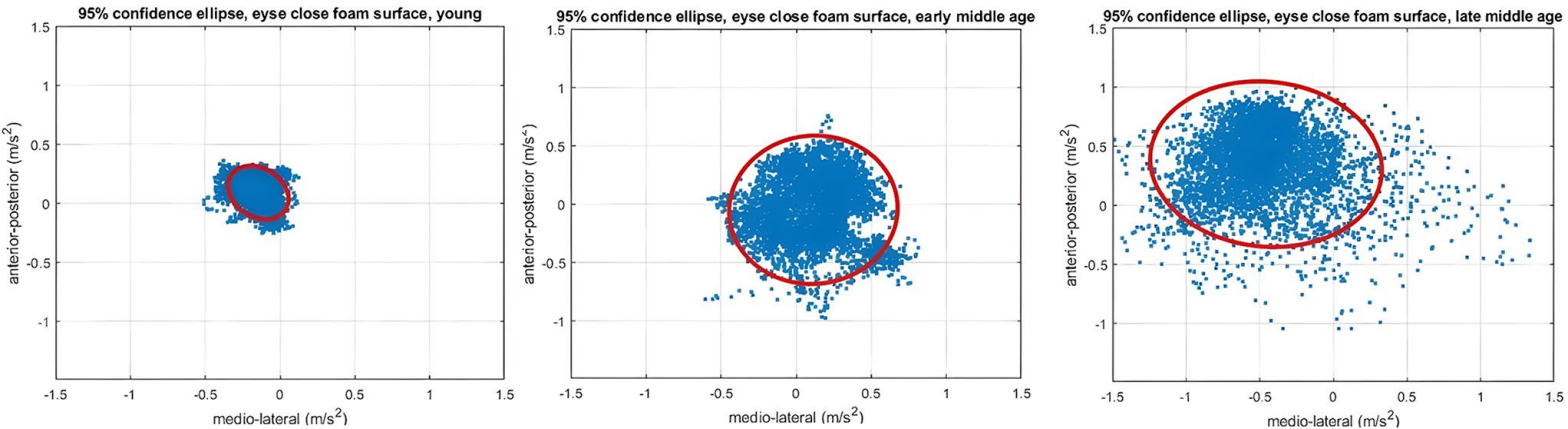
Example of the COM-95% ellipse of one subject in each group during the Sensory-Orientation test. COM- center of mass, 95% ellipse- 95% Confidence ellipse

A summary of the accelerometry data and their comparisons between groups are presented in Table 2. Significant between-group differences were observed in the COM-95% ellipse recorded during both instrumented Brief-BESTest measures. Specifically, significant differences were found in the COM-95% ellipse size of the Anticipatory-Postural-Adjustments for both the right (*X*^*2*^ [2] = 6.45, *p* = 0.04) and left (*X*^*2*^ [2] = 6.14, *p* = 0.04) legs. Post hoc analysis revealed a larger COM-95% ellipse size in LMA compared with YA and in both right and left legs. In addition, significant between groups differences were found in COM-95% ellipse of the Sensory-Orientation (X2 [2] = 12.36, *p* = 0.002; see Figs. 2 and 3). Post hoc analysis revealed larger COM-95% ellipse size in both, EMA and LMA groups compared to YA (*p* = 0.019, *p* = 0.003, respectively). As the COM-95% ellipse of the Sensory Orientation differed between both middle-aged groups to YA, it was further analyzed with variance testing to identify middle-aged subjects in the poor performance quartile.

**Table 2:** Balance evaluation scores- Brief-BESTest total and sub-measure scores, and the COM-95% ellipse data

Measurements	Young (*n* = 25)	Early Middle age (*n* = 25)	Late Middle age (*n* = 25)
**Brief-BESTest total score (24)**	24 (24–24) ^**a b**^	22 (20.5–23) ^**c**^	20 (18–21)
**Biomechanical-Constraints (3)**	3 (3–3) ^**a b**^	3 (2–3) ^**c**^	2 (2–2)
**Stability-Limits (3)**	3 (3–3) ^**a b**^	3 (2–3)	2 (2–2)
**Anticipatory-Postural-Adjustments (6)**	6 (6–6) ^**a b**^	6 (5–6)	5 (4–6)
**Reactive-Postural-Responses (6)**	6 (6–6) ^**a b**^	6 (5–6)	6 (5–6)
**Sensory-Orientation (3)**	3 (3–3) ^**b**^	3 (2–3) ^**c**^	2 (2-2.5)
Stability-in-Gait (3)	3 (3–3)	3 (3–3)	3 (3–3)
**Instrumented Measures**			
**Anticipatory-Postural-Adjustments Right** **COM-95% ellipse (** $\:{\varvec{m}}^{4}\cdot\:{\varvec{s}}^{-2})$	0.23 (0.17–0.39) ^**b**^	0.31 (0.15–0.62)	0.49 (0.29–0.68)
**Anticipatory-Postural-Adjustments Left** **COM-95% ellipse (** $\:{\varvec{m}}^{4}\cdot\:{\varvec{s}}^{-2})$	0.24 (0.14–0.46) ^**b**^	0.25 (0.19-1.00)	0.48 (0.25–0.87)
**Sensory-Orientation COM-95% ellipse (** $\:{\varvec{m}}^{4}\cdot\:{\varvec{s}}^{-2})$	0.29 (0.25–0.37) ^**a b**^	0.40 (0.33–0.63)	0.50 (0.32–0.76)

Median (25-75th percentile interquartile range)

- All comparisons were conducted using Kruskal-Wallis tests

- All *p* values are with Bonferroni adjustment for multiple comparisons

^**a**^ Significant differences between the young and early middle-aged groups (*p* < 0.05)

^**b**^ Significant differences between the young and late middle-aged groups (*p* < 0.05)

^**c**^ Significant differences between the early and late middle-aged groups (*p* < 0.05)

COM- center of mass, 95% ellipse- 95% Confidence ellipse

The box plots showing the distribution across groups in the Brief-BESTest total score and the Sensory Orientation COM-95% ellipse are presented in Figs. 1 and 2, respectively. The division into quartiles revealed middle-aged subjects in the poor-performance quartile. This included six EMA participants and seven LMA participants in the poor performance quartile of the Brief-BESTest total score and six EMA participants and seven LMA participants in the poor performance quartile of the COM-95% ellipse of the Sensory Orientation.

The Kappa which tested the agreement between the Brief-BESTest total score and the COM-95% ellipse recorded during Sensory Orientation in identifying subjects with poor performance yielded a moderate agreement (*k* = 0.56, *p* = 0.005) in the EMA group. Conversely, no significant agreement between these tests was found in the LMA group. Additionally, a significant moderate Spearman negative correlation (*r* = -0.43, *p* < 0.001) was found between the Brief-BESTest’s total score and the COM-95% ellipse of the Sensory Orientation in all three groups.

Due to between-group differences in the participants’ background characteristics, a pre-planned sensitivity analysis using manual step linear regression was deployed on the main balance outcomes, the Brief-BESTest total score (which is the sum of all Brief-BESTest sub-measures), and the COM-95% ellipse of the Sensory Orientation task. A significant regression was found for the Brief-BestTEST total score (f [2,72] = 36.91, *p* < 0.001), with r2 = 0.49. The addition of background characteristics as covariates did not increase the explained variance. A significant regression was found for the COM 95% ellipse of sensory orientation (f [2,70] = 3.15, *p* = 0.04), with r2 = 0.05. The addition of background characteristics as covariates did not increase the explained variance. The stepwise linear regression is shown in the supplementary data, Table S3. An additional regression model examining the effect of age group on the main balance tests showed that both the EMA and LMA groups had significantly lower Brief-BESTest total scores and a higher COM 95% ellipse of sensory orientation compared to the YA reference group. The linear regression is shown in the supplementary data, Table S4.

## Discussion

The aim of this study was to evaluate the potential of the Brief-BESTest and instrumental assessment of sub-measures of the Brief-BESTest in detecting balance deterioration in middle-age and identifying middle-aged individuals with poor balance. Our results suggest that both the Brief-BESTest and the partially instrumental assessment of the Brief-BESTest can discriminate between young and middle-aged adults. Furthermore, the Brief-BESTest total score differed also between early and late middle-aged individuals (i.e., 45–54 years; 55–65 years). These results demonstrate the utility of these tests in detecting early deterioration of balance in middle age and provide practical tools to support the existing evidence regarding the benefits and necessity of balance testing before the age of 65 [8–10].

Our findings are consistent with those of O’Hoski et al. [34], who showed a significant decrease in Brief-BESTest total score in healthy adults aged 50 to 89 years. Here, we extend these findings by showing that balance decreases in middle-aged compared to young adults. Furthermore, in agreement with recent studies emphasizing the importance of examining performance within subgroups in midlife to better understand aging [23–25, 34, 49, 50], our results also show a decline in balance abilities in late compared to early midlife. Although other balance tests, such as the Balance Error Scoring System [51, 52] and the Y-Balance Test [53, 54], have also shown age-related deterioration of balance in middle age, the Brief-BESTest is a much more comprehensive test that covers six balance systems and therefore appears to be better suited to detect early deterioration of balance in the middle-aged population.

The increased variance in balance ability with age allowed us to identify middle-aged individuals with poor performance. O’Hoski et al. [34], suggested that visual inspection of box plots could indicate an increase in variation in balance with age. Our findings also suggest that the variance in balance performance increases with age. The large variation in balance performance among middle-aged individuals assisted us in identifying individuals with accelerated balance deterioration who were defined as those in the poor performance quartile. The poor performance quartile threshold for the Brief-BESTest total score was < 20.5 and < 18 points, for early and late middle age respectively, and the total scores of subjects included in the poor performance quartile were similar to those of older adults a decade older [34, 55]. This could indicate a discrepancy between the chronological and biological age of the subjects in the quartile with poor performance in terms of their balance function and may explain the reports of reduced mobility and the incidence of falls in this age group [12–16, 56, 57]. While our results suggest that balance assessment with the Brief-BESTest may facilitate early detection of balance deterioration in middle age, future studies with larger samples should further establish the utility of the Brief-BESTest for this purpose.

The analysis of the sub-measures of the Brief-BESTest revealed that all sub-measures except Stability-in-Gait (Timed Up and Go) differentiated between young and middle-aged adults. In agreement with O’Hoski et al. [34], all subjects in the middle-aged groups achieved the maximum score on the Timed Up and Go test, which may indicate that the Timed Up and Go test may not be sensitive enough to detect balance deterioration in the middle-aged population. Yet, only the Biomechanical-Constraints and Sensory Orientation sub-measures differed between early and late middle-aged individuals. The Biomechanical-Constraints represent the mechanical properties of balance (i.e., range of motion and strength) [29] and it is tested by evaluating hip/trunk lateral strength [30]. Our results that demonstrated a reduction in hip/trunk strength in late compared to early middle-aged individuals add to the evidence regarding strength decline during middle age. Lindle et al. [23], found that strength begins to decline in the fourth decade, and Bohannon et al. [24, 25], reported a decline in 5-repetition sit-to-stand test performance beginning in the fifth decade of life. The Sensory Orientation sub-measure assesses the body sway during stance on foam with eyes closed [30] which is associated with the function of the visual and somatosensory systems [29]. Our results are consistent with Springer et al. [50], who reported a reduction in single-leg stance duration with eyes closed between 40 and 49 and 60–69 years and between 50 and 59 and 60–69 years. This may suggest that the balance regulatory systems tested by the biomechanical constraints and the sensory orientation sub-measures deteriorate at an accelerated rate in middle age. Further investigation is recommended to determine whether additional balance measures can differentiate between middle-aged individuals.

In this study, the standing tasks of the Brief-BESTest (i.e., the one-legged stance and standing with eyes closed on a foam surface) were also measured with an accelerometer to calculate the 95% ellipse size of the COM trajectory. Our results show that the COM-95% ellipse, representing body sway, was larger in the late middle-aged compared to young adults in both standing conditions, and in the early middle-aged compared to young adults when standing on a foam surface with eyes closed. Increased body sway during standing tasks indicates decreased balance and has been considered a potential risk factor for falls [58–60]. Other studies have used the COM-95% ellipse to assess balance in healthy and unhealthy middle-aged and older adults [61–68]. However, to our knowledge, this is the first study that used the COM-95% ellipse to test balance in healthy middle-aged adults and compare them with young adults.

By analyzing the COM-95% ellipse size data we were also able to identify middle-aged individuals in the poor performance quartile. A moderate negative correlation (*r* = -0.43) was found between the Brief-BESTest total score and the COM-95% ellipse size. In addition, there was moderate agreement (*k* = 0.56) between the Brief-BESTest and the COM- 95% ellipse in identifying subjects with poor performance in the early but not the late middle age group. The lack of agreement in identifying individuals with poor balance in the late middle age group by these two outcomes and the only moderate agreement in the early middle age group may suggest that considering the results of the two tests could provide a more comprehensive assessment of an individual’s balance abilities. Unlike the Brief-BESTest, which is scored by a clinician, an accurate measurement of sway cannot be made without appropriate measuring equipment. Furthermore, each test may capture different aspects or nuances of balance, therefore, providing a more complete picture which may allow a better identification of individuals with poor balance. However, it is also recommended that future research will investigate factors that may contribute to the discrepancies between the tests. It should also be noted that sway assessment such as the size of the COM-95% ellipse can be measured using smartphones, which are equipped with accelerometers and are inexpensive, making this measurement simple and more accessible.

Like all research, this study is not without limitations. The first limitations are related to the cohort studied. The study cohort consisted of students and university employees volunteers who may have certain characteristics compared to the non-volunteer population [69]. Although the sample size in each group was larger than in a previous similar study [34], the study sample size may not be sufficient to characterize the middle-aged population. In addition, we only included participants who stated that they were able to perform moderate to vigorous physical activities of daily living. More rigorous screening could have included additional and more diverse healthy middle-aged subjects who might perform differently on the balance tests. A second limitation of the present study pertains to factors that may influence balance. Our balance assessment results were not influenced by lifestyle and general health factors such as BMI, total strength (measured by maximal grip strength), and physical activity level (measured by the IPAQ-SF). Yet, further studies should investigate the influence of additional lifestyle and general health factors on balance during middle age. Finally, the accelerometry assessment of the Brief-BestTEST only included the stationary tasks. It is recommended that further research assess the utility of instrumenting all Brief-BestTEST tasks in identifying middle-aged individuals with early balance deterioration.

## Conclusions

Recognizing deterioration in balance and identifying individuals with poor balance before the age of 65 has great potential for improving clinical practice, as it can enable the implementation of preventive strategies before the risk increases. While common clinical balance tests have a ceiling effect even in community-dwelling older adults, the present findings show the utility of the Brief-BESTest partially combined with accelerometry in detecting early balance decline during middle age and particularly in identifying middle-aged individuals with poor balance and possibly higher risk for falls. These results can serve as a basis for further studies that will test the clinical applicability of this measure and its ability to predict balance and mobility performance in older age.

## Electronic Supplementary Material

Below is the link to the electronic supplementary material.

Supplementary Material 1

## Data Availability

Data is provided within the manuscript and supplementary information files.
