# Supplementary material for: Use of the Brief-BESTest partially instrumented with accelerometry to detect balance deterioration in middle-age
Source: Aging Clin Exp Res. 2024 Nov 9;36(1):214. doi: 10.1007/s40520-024-02868-7 (PMC11550257; doi:10.1007/s40520-024-02868-7)
Supplement: Supplementary file 1 — Supplementary Material 1 [file 40520_2024_2868_MOESM1_ESM.docx]

**Table 3:** Step linear regression for the effect of possible background covariates on Brief-BESTest total score and the COM-95% ellipse of the Sensory-Orientation.

| Model Predictors | Brief-BESTest total score | | COM-95% ellipse, Sensory-Orientation | |
| --- | --- | --- | --- | --- |
|  | Adjusted *r^2^* | Sig *F* change | Adjusted *r^2^* | Sig *F* change |
| Group | **0.49** | ***p* < 0.001** | **0.05** | ***p =* 0.04** |
| Group and BMI | 0.50 | *p* = 0.06 | 0.08 | *p =* 0.09 |
| Group, BMI, and grip strength | 0.51 | *p* = 0.25 | 0.09 | *p =* 0.12 |
| Group, BMI, grip strength and physical activity | 0.51 | *p* = 0.31 | 0.08 | *p =* 0.56 |

BMI- Body mass index, COM- center of mass, 95% ellipse- 95% Confidence ellipse.

**Table 4**: Linear regression presenting the effect of age group on the Brief-BESTest total score and the COM-95% ellipse of the Sensory-Orientation.

|  | Brief-BESTest total score | | COM-95% ellipse, Sensory-Orientation | |
| --- | --- | --- | --- | --- |
|  | Coefficient | p-value | Coefficient | p-value |
| Group **^a^**: EMA | -2.16 | ***p* < 0.001** | 0.26 | ***p* = 0.03** |
| Group **^a^**: LMA | -4.20 | ***p* < 0.001** | 0.26 | ***p* = 0.03** |

**^a^** Young adults group was set as a reference.

EMA- Early middle age, LMA- Late middle age, COM- center of mass, 95% ellipse- 95% Confidence ellipse
